# Multimodal Imaging Evaluation and Follow-up of a Congenital Left Ventricular Aneurysm

**DOI:** 10.1016/j.cjco.2025.06.002

**Published:** 2025-06-09

**Authors:** Matteo Brusamolino, Ruper Oliveró, Victor González, Jose F. Rodriguez-Palomares, Gianluca Pontone

**Affiliations:** a Department of Perioperative Cardiology and Cardiovascular Imaging, Centro Cardiologico Monzino IRCCS, Milan, Italy; b University of Milan, Milan, Italy; c Echocardiography and Cardiac Imaging Unit, Cardiology Department, University Hospital Vall d’Hebron, Barcelona, Spain; d Adolescent and Adult Congenital Heart Disease Unit, Cardiology Department, University Hospital Vall d’Hebron, Barcelona, Spain; e Cardiac Imaging Unit, Aortic Diseases and Inherited Cardiomyopathies, Cardiology Department, University Hospital Vall d’Hebron, Barcelona, Spain; f European Reference Network for Rare and Low Prevalence Complex Diseases of the Heart, ERN GUARD-Heart, Amsterdam, The Netherlands; g Centro de Investigación Biomédica en Red en Enfermedades Cardiovasculares (CIBERCV), Madrid, Spain; h Department of Biomedical Surgical and Dental Sciences, University of Milan, Milan, Italy

**Keywords:** cardiovascular imaging, left ventricular aneurysm, cardiovascular computed tomography, echocardiography, cardiovascular magnetic resonance

**Congenital left ventricular aneurysm (CLVA) is a rare cardiac malformation with an unknown etiopathogenesis and variable clinical manifestations. Due to the rarity of this condition, no established guidelines exist, and clinical management must be individualized. We report a case of an incidental diagnosis of a CLVA in a 37-year-old man, with a 4-year follow-up. We highlight the role of noninvasive multimodal cardiac imaging in supporting diagnosis, enabling periodic monitoring, and excluding potential complications associated with this disease. In addition, we demonstrate how a watchful “wait and see” approach may be applicable in this rare condition.**

## Case Presentation

A 37-year-old asymptomatic Caucasian male patient was referred for a cardiological evaluation due to electrocardiographic (ECG) repolarization abnormalities in the anterolateral leads, identified during a preoperative assessment for orthopedic surgery. The patient was an active smoker and reported no significant personal or family medical history.

**Figure 1:**
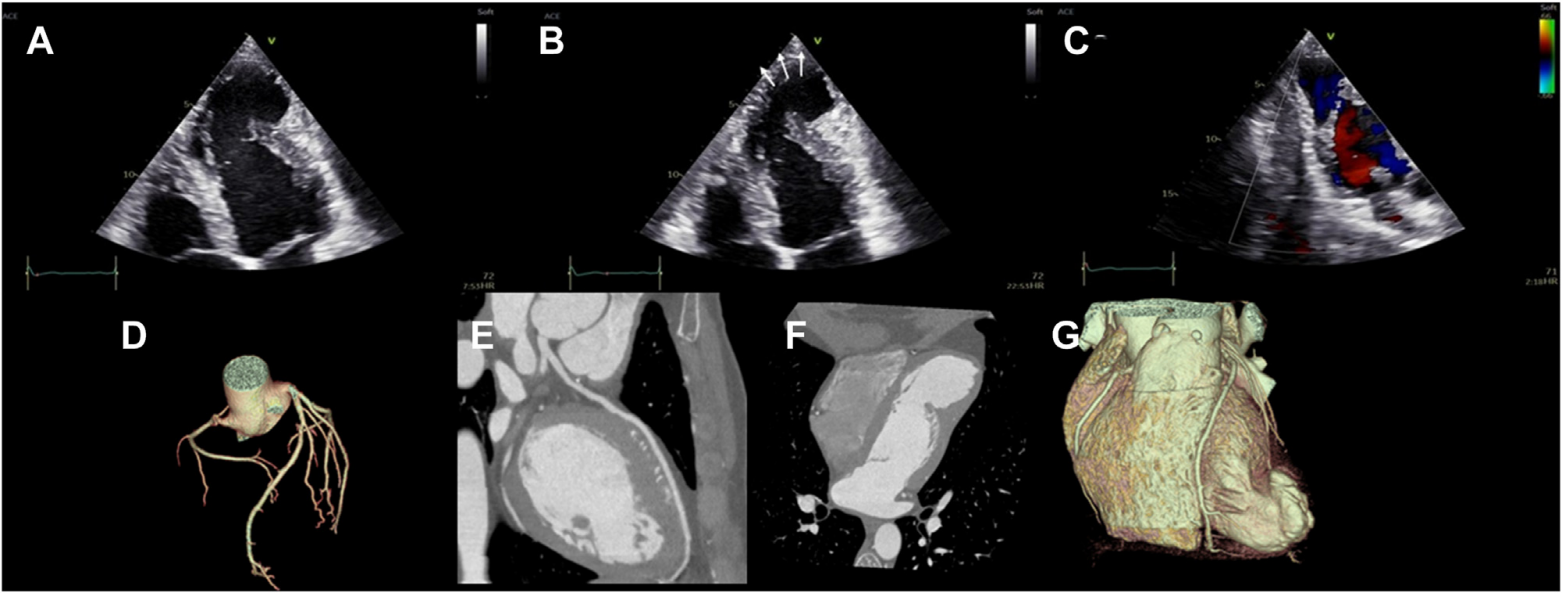
Transthoracic echocardiography and cardiac computed tomography. Long-axis 4-chamber view of the (**A**) diastolic frame and (**B**) systolic frame showing a left ventricular (LV) apical aneurysm with a wide neck connecting it to the LV cavity. **Arrows** indicate dyskinetic wall motion (systolic paradoxical expansion). (**C**) Color Doppler imaging shows no flow acceleration across the aneurysmal neck. (**D**) Computed tomography coronary 3-dimensional reconstruction showing normal origin and course of epicardial coronary arteries. (**E**) Absence of left anterior descending coronary artery stenosis is demonstrated. (**F**) Reformatted apical view and (**G**) volume-rendered 3-dimensional reconstruction showing the LV aneurysm.

His physical examination was unremarkable. Results of routine blood tests, including brain natriuretic peptide levels, were within normal limits. Transthoracic echocardiography (Fig. 1; Video 1, view video online) revealed a large left ventricular (LV) apical aneurysm with dyskinetic wall motion and a wide neck connecting it to the ventricular cavity, with no evidence of color flow or pulsed-wave Doppler acceleration. No intraventricular thrombus was detected. To exclude an ischemic etiology, coronary computed tomography angiography (CCTA) was performed. The CCTA revealed normal coronary origin and course, yet a paucity of coronary distribution to the aneurysm, and mild coronary atherosclerosis without any relevant stenosis. The wide LV apical aneurysm was confirmed (Fig. 1). To evaluate the potential for arrhythmic events, 24-hour ambulatory electrocardiographic monitoring was performed, revealing no significant arrhythmias. A maximal symptom-limited exercise stress test was negative for ischemic ST-segment changes and showed no exercise-induced arrhythmias. Cardiac magnetic resonance (CMR) imaging showed mild LV dilatation with preserved systolic function (LV end-diastolic volume index, 111 mL/m^2^; LV ejection fraction, 53%) and an LV apical aneurysmal cavity (52 x 43 x 43 mm) with dyskinetic wall motion. A thin layer of subendocardial late gadolinium enhancement of the aneurysmal wall was observed. No intraventricular thrombus or associated congenital cardiac anomaly was detected.

An ischemic etiology was excluded based on normal coronary findings on CCTA and CMR imaging features. Other causes of LV aneurysm—including cardiomyopathies (such as hypertrophic obstructive cardiomyopathy with midventricular obstruction, and arrhythmogenic cardiomyopathy), excessive trabeculation of the LV, Chagas disease, myocarditis, and sarcoidosis—were ruled out through CMR imaging findings, clinical history, and the absence of systemic features of autoimmune disease or markers of systemic infection. A diagnosis of CLVA was made. Given the size of the aneurysm and the potential thromboembolic risk, the patient was eventually started on rivaroxaban 20 mg once daily. Bisoprolol 2.5 mg once daily was prescribed for neurohormonal modulation and antiarrhythmic effect. We advised against high-intensity, contact, power, and isometric activities, due to their high cardiovascular impact. Periodic clinical follow-up was performed.

**Figure 2:**
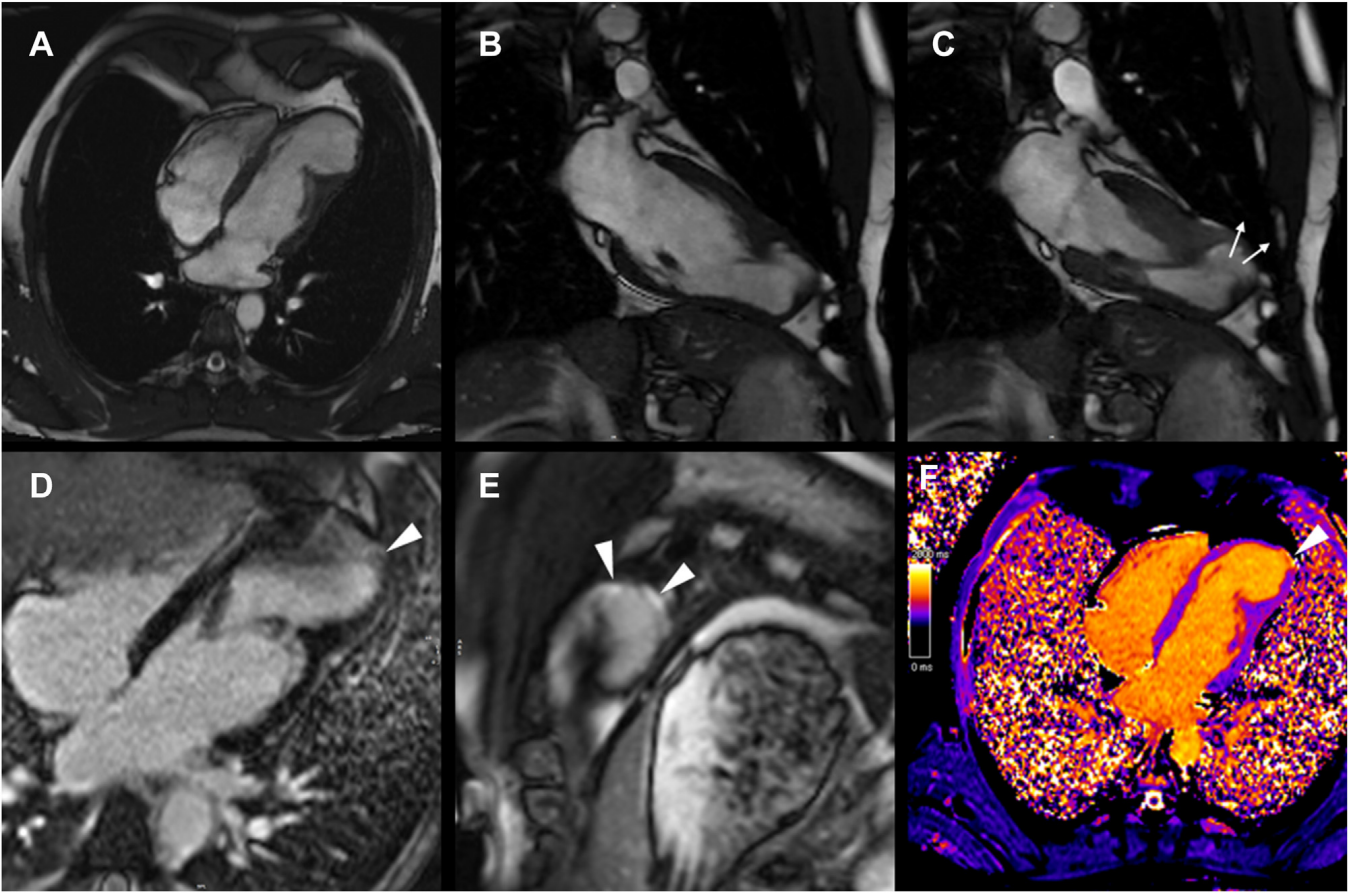
Cardiac magnetic resonance (CMR) images. (**A**) Long-axis 4-chamber view, diastolic frame, and (**B, C**) long-axis 2-chamber view, diastolic and systolic frames, showing a large left ventricular apical aneurysm. **Arrows** in (**C**) indicate dyskinetic wall motion. (**D**) Long-axis 4-chamber view and (**E**) apical short-axis view late gadolinium enhancement images showing fibrosis (**arrowheads**) of the thin aneurysmal wall. (**F**) Long-axis 4-chamber T1-mapping image showing normal myocardial native T1 values, with focal increase at the level of the aneurysmal wall (**arrowhead**), where late gadolinium enhancement was observed.

Four years later, the patient remains asymptomatic. He has quit smoking, has an active lifestyle, and reports having independently discontinued rivaroxaban. His brain natriuretic peptide level is 64 pg/mL. No arrhythmias were detected during periodic monitoring, and no significant arrhythmia-related symptoms were reported. CMR imaging (Fig. 2; Video 2, view video online) showed mild LV dilatation (LV end-diastolic volume index, 108 mL/m^2^) with preserved systolic function (LV ejection fraction, 54%). The dimensions of the apical aneurysm remained stable (54 x 44 x 43 mm, measured in horizontal long-axis breath-hold balanced steady-state free precession cine sequences), and no intraventricular thrombus was detected. The aneurysmal wall exhibited late and dyskinetic wall motion compared to the rest of the ventricle. A thin layer of subendocardial late gadolinium enhancement was confirmed, with a corresponding increase in native T1 values. Normal T2 values were detected on T2-mapping sequences. No abnormalities in the pericardium were noted. All the other findings were within normal limits.

Given the patient's stable condition, annual clinical follow-up was recommended, including ECG and echocardiography, along with periodic Holter monitoring and exercise stress testing to assess for arrhythmias. Advanced imaging with CMR or cardiac computed tomography will be considered if any changes are detected on transthoracic echocardiography. Closer clinical and imaging monitoring will be required should symptoms or signs of structural disease progression arise.

## Discussion

CLVA is a rare and poorly understood cardiac malformation, with a prevalence of up to 0.34% in some studies.^1^ These apical outpouchings are located most commonly in the submitral region and often are associated with mitral valve disease. They may present as isolated findings or with other cardiac and vascular anomalies, including ventricular and atrial septal defects, coronary artery anomalies, mitral or aortic valve dysfunction, and aortic coarctation. In some cases, they may be associated with extracardiac anomalies, such as thoracoabdominal wall defects.^2^ CLVAs are thought to originate as developmental defects during the 4th week of embryonic gestation,^3^ without a clear genotype-phenotype correlation. Although most cases are asymptomatic and often are discovered incidentally, symptomatic cases may present severe and potentially life-threatening manifestations. In the population described by Ohlow et al., ventricular tachyarrhythmic events occurred at presentation in 18.1% of cases, syncope in 8.3%, rupture in 4%, and embolic events in 4.9%, whereas thrombotic material was present within the aneurysms in 11% of cases.^2^

Regarding the etiology, coronary artery disease was excluded based on the normal coronary findings on CCTA. Other potential etiologies (such as cardiomyopathies, myocarditis, sarcoidosis, chest trauma, congenital partial absence of the pericardium with ventricular herniation) were ruled out based on CMR imaging findings, clinical history, and the absence of systemic features of autoimmune disease or systemic infectious markers.^3^^,^^4^ CLVA must be differentiated from LV diverticula (LVD).^3^ LVD is characterized by a finger- or hook-like appendix, contracting in synchrony with the corresponding chamber with a ratio < 1 of the connection to the body of the anomaly. On the other hand, CLVA is an akinetic or dyskinetic structure with a wide connection to the left ventricle (ratio > 1), as demonstrated in our patient. A systematic analysis by Ohlow et al. provided key insights into the clinical course of patients with CLVA and LVD.^2^ Among 205 CLVA patients with available follow-up (mean duration, 62.8 ± 48.3 months), arrhythmic and embolic events occurred in 23 and 2 patients, respectively. Cardiac death was reported in 12.7% of cases, primarily due to heart failure (50%), sudden cardiac death (26.9%), and rupture (23.1%). Overall, cardiac death was significantly more frequent in the CLVA group, and event-free survival during follow-up was significantly lower in the CLVA group than in the LVD group.

Our findings illustrate how various imaging modalities can be integrated to support diagnosis, evaluate disease progression, inform clinical decision-making, and identify potential complications over time. Echocardiography is the primary tool for assessing LV size and function, and it is therefore likely to be the first investigation to identify cardiac outpouchings. It may assist in detecting congenital abnormalities associated with CLVA, such as valvular disease or intracardiac shunts. Additionally, echocardiography can reveal mural thrombi, with use of contrast agents if necessary. However, its utility may be limited by poor acoustic windows in certain patients and by operator dependency. CCTA has been demonstrated to be a safe, rapid, and effective noninvasive technique for ruling out coronary artery disease in patients with a low-to-intermediate risk. Moreover, CCTA enables the exclusion of coronary artery anomalies and vascular abnormalities that may be associated with this condition. Additionally, multiplanar and volume-rendered CT reconstructions provide excellent 3-dimensional visualization of LV aneurysm. CMR imaging is the gold standard for assessing myocardial function, volumes, and tissue characterization. CMR imaging is highly effective for visualizing LV outpouchings, identifying wall-motion abnormalities, and detecting LV thrombus. Additionally, CMR imaging may aid in differential diagnosis. CMR imaging is essential for long-term follow-up, as it allows for the assessment of aneurysmal size and LV volumes and function over time.

Management should be tailored to the patient’s clinical presentation and specific findings. An initial conservative management approach was adopted in view of the lack of symptoms. Despite the paucity of evidence supporting its use, the patient was started on anti-remodelling and antiarrhythmic therapy with a beta-blocker, and prophylactic anticoagulation with rivaroxaban. Given the absence of significant arrhythmias and no reported history of syncope, we opted not to implant an implantable cardioverter defibrillator for primary prevention. As the patient did not report chest pain or equivalent symptoms, noninvasive functional imaging testing for ischemia detection was not performed. The decision to refrain from escalating medical therapy or recommending surgical resection was supported by a comprehensive assessment integrating clinical evaluation and imaging findings (the absence of heart failure signs or symptoms, the lack of arrhythmias or related symptoms, the absence of thromboembolic complications, the stability of LV dimensions and function, the stable size of the aneurysm over time, and the lack of associated congenital anomalies necessitating surgical intervention).

We presented a case of multi-year follow-up of a CLVA, which is a rare congenital cardiac abnormality that may be associated with catastrophic complications. Multimodal noninvasive cardiac imaging techniques, perceived as complementary imaging approaches, play an essential role in the evaluation of patients with this disease.

**Novel Teaching Points**

• CLVA is a rare cardiac malformation with a highly variable clinical presentation.
• Ruling out other potential causes of aneurysm formation is essential to confirm the CLVA diagnosis.
• Due to the absence of established management guidelines, CLVA treatment strategies are customized based on the patient's clinical presentation and specific findings.
• Multimodal noninvasive cardiac imaging supports CLVA diagnosis, enables periodic monitoring, and informs clinical decision-making.

## Supplementary Material

Video 1 Transthoracic Echocardiography (TTE) Apical Views. TTE apical views showing a large LV apical aneurysm. Dyskinetic wall motion and the wide neck are clearly appreciable. Color Doppler imaging shows no flow acceleration across the aneurysmal neck. No intraventricular thrombus is observed.

Video 2 Cardiac Magnetic Resonance (CMR) Cine Sequences Balanced steady-state free precession (bSSFP) CMR Cine sequences in long-axis 4-chamber and 2-chamber view provide optimal visualization of the LV apical aneurysm. Dyskinetic wall motion is clearly appreciable using this imaging technique
